# Anaerobic threshold, is it a magic number to determine fitness for surgery?

**DOI:** 10.1186/2047-0525-2-2

**Published:** 2013-02-21

**Authors:** Paul Older

**Affiliations:** 1 Department of Anaesthetics, Western Hospital, Melbourne, Australia

## Abstract

The use of cardiopulmonary exercise testing (CPET) to evaluate cardiac and respiratory function was pioneered as part of preoperative assessment in the mid 1990s. Surgical procedures have changed since then. The patient population may have aged; however, the physiology has remained the same. The use of an accurate physiological evaluation remains as germane today as it was then. Certainly no ‘magic’ is involved. The author recognizes that not everyone accepts the classical theories of the anaerobic threshold (AT) and that there is some discussion around lactate and exercise. The article looks at aerobic capacity as an important predictor of perioperative mortality and also looks at some aspects of CPET relative to surgical risk evaluation.

## Review

### The ‘magic’ of it all!

The word ‘magic’ conjures up visions of Merlin, Camelot and mystical beasts around 1100 AD. This was some 600 years before the real magic, that of Lavoisier, was initiated. Antoine Laurent Lavoisier was beheaded in 1794 during the French Revolution but not before he had measured oxygen uptake during exercise. This provided the fundamental foundation for modern CPET.

Lavoisier showed that during respiration oxygen is consumed and carbon dioxide is given off. At the time of Lavoisier’s work, chemistry was so underdeveloped that it could hardly be called a science. At the beginning of the eighteenth century, chemistry was alchemy; by the end it was a science. For more detail on Lavoisier, the reader is referred to references [1,2].

Far from being magic, the measurement of oxygen uptake and the traditional exposition of the anaerobic threshold (AT) is the result of a logical physiological progression [3]. It is the epitome of *Vorsprung durch Technik*. This slogan, *Progress through technology*, has been used by Audi AG since 1985. Although a cliché, it encapsulates the author’s perception of cardiopulmonary exercise testing. So many famous names have pursued the concepts that underlie this physiology: Lavoisier, Krogh, Bohr, Henderson, Dill, Wasserman, Whipp, Jones, to name a few.

A series of works published back-to-back in one journal in 1910 by August Krogh, a Nobel Laureate in 1920, (‘The seven little devils’) [4] attracted special attention because he demonstrated that ‘the absorption of oxygen and elimination of carbon dioxide in the lungs take place by diffusion and by diffusion alone. There is no trustworthy evidence of any regulation of this process on the part of the organism’ [5]. This statement was in direct conflict with the position of Christian Bohr, who believed there to be an active transport mechanism for the transport of oxygen across the alveoli. (For a detailed discussion on the disagreement between these two titans, see [6]). Krogh had also developed a cycle ergometer with which he was able to measure insensible and sensible fluid losses accurately during heavy exercise [5].

In 1914, Yandell Henderson named the oxygen pulse as a most important quantitative measurement. It was defined as ‘the oxygen extraction of one systolic discharge’ [7]. Henderson went on in 1923 to state, ‘The stroke volume of the heart is for physiological and clinical purposes the most important quantitative function of the whole body’ [8].

The Harvard Fatigue Laboratory, a laboratory of human physiology, was conceived in 1926 by LJ Henderson and began operation in 1927. The staff of the Harvard Fatigue Laboratory embraced a wide range of disciplines, including physiology, biochemistry, psychology, biology, medicine, sociology and anthropology. The research performed by the laboratory reflected this diversity of backgrounds; areas of research included the physical chemistry of blood, exercise physiology, nutritional interactions, aging, and the stresses of high altitude and climate. Equipment utilized by the staff in conducting research included treadmills, a climatic room, an altitude chamber and an animal room. The physiologist DB Dill was the director of the laboratory from 1927 to 1946. This laboratory was responsible for innumerable publications relating to exercise physiology [9].

In 1960, Julius Comroe, the Director of the Cardiovascular Research Institute at the University of California in San Francisco where Wasserman was working, pointed out to him that heart disease in the USA was increasing in epidemic proportions. Comroe asked Wasserman how patients could be screened, noninvasively if possible, for early detection of heart failure and other heart diseases (K Wasserman, personal communication). In 1963, Naimark, Wasserman and McIlroy showed that exercise tests could usefully be employed to detect degrees of dysfunction in the cardiovascular system. The onset of anaerobic metabolism, the AT, during exercise could be detected in three ways; an increase in lactate concentration in the blood, a decrease in arterial bicarbonate concentration and pH, and an increase in the respiratory exchange ratio [10,11]. Wasserman showed that the lactic acid must be buffered, by the volatile buffer bicarbonate, producing an equal number of CO_2_ molecules to be added to the CO_2_ produced by aerobic metabolism.

Since rapidly responding gas analyzers had recently become available, Wasserman further suggested that the CO_2_ produced by the bicarbonate buffering of lactic acid might be measured breath-by-breath.

Comroe then suggested that Wasserman should pursue these ideas (K Wasserman, personal communication). His initial studies on the physiological underpinnings of this work were with Naimark and McIlroy. They showed how the respiratory exchange ratio could be measured on a continuous basis during exercise studies. Further, they showed that the increase in the respiratory exchange ratio was related to work intensity [10].

Their first work on the AT was published in 1964 from Stanford [11]. In 1967, Dr Wasserman was invited by UCLA to become Division Chief at Harbor General Hospital, later Harbor-UCLA Medical Center. Brian J Whipp, then a predoctoral Fellow with Wasserman at Stanford, moved with him: together they set up the exercise physiology research laboratory and collaborated in exercise physiology research.

With the development of digital computers later in the 1960s, WL Beaver, a physicist working at Central Research at Varian Associates in Palo Alto, joined this investigation. This collaboration enabled the use of digital computers for breath-by-breath exercise gas exchange [12-14]; *Vorsprung durch Technik*?

Wasserman, with colleagues such as Brian Whipp, James Hansen, Daryl Sue, Kathy Sietsema, Dan Cooper, Richard Casaburi and William Stringer, became the driving force behind the development of cardiopulmonary exercise testing in the clinical environment. To this day, they still publish key literature on the information gained about cardiac and respiratory function from cardiopulmonary exercise testing. Many other names deserve mention but the space available precludes that.

Enough has been said to deny the use of the term ‘magic number’ to describe any part of the physiological evolution that describes cardiopulmonary exercise testing.

### The anaerobic threshold (AT)

To turn to CPET, the term anaerobic threshold (AT) embraces two quite separate issues. Is there a point that defines ‘anaerobic’ and precisely what is meant by ‘anaerobic’? Is there a threshold? The reader is referred to a masterly article by Whipp in 2008, ‘The anaerobic threshold: yes, no, maybe!’ [15]. If in 2008 there was still controversy over the term, then that alone warrants further examination of just why.

The latest book by Wasserman *et al.*[16] could be used with advantage to obtain a detailed description of determination of the AT.

One issue that remains is the inter-rater and intra-rater repeatability of the data measurements, in particular the AT. This is impossible to quantify as it is related directly to the experience of the physicians. In our laboratory it is uncommon, or rare, for there to be a significant disagreement.

### Lactate

Anaerobic metabolism is not uniquely a feature of high intensity work rates; on the contrary it is found at low intensity work rates but does not represent a major component of energy transfer under those conditions. It is probable that there is a degree of anaerobic metabolism at all work rates [17]. Certainly at the onset of exercise there is an increase in ATP demand. This is met from oxygen independent sources, such as phosphocreatine hydrolysis and glycolysis [18]. This occurs in the presence of normal tissue oxygenation but does not, in the author’s opinion, constitute anaerobic metabolism in the sense in which it is commonly used in clinical medicine. Further lactate, the most commonly quoted suspect as the end point of anaerobic metabolism, is falsely accused. Lactate is a product in the pathway of energy production, not its endpoint. Lactate is produced by muscle tissue at rest in the absence of any cause of hypoxia [19]. It was thought that lactate at the end of exercise was converted back to glycogen *in situ*, thus requiring oxygen, which was termed the ‘oxygen debt’. It is now recognized that lactate present at the end of exercise is predominately oxidized [20]. To open this Pandora’s box, the reader is directed to the classic reviews of lactate metabolism by Gladden [19] and by Stainsby [21]. The term ‘anaerobic threshold’ relates to a point during increasing exercise where the increase in concentration of lactate is accompanied by an almost equal reduction in the concentration of bicarbonate. Put another way, it is the highest work rate at which the concentration of lactate does not show a consistent increase. The latter is accompanied by an increase in CO_2_ output, a phenomenon that was used by Beaver and colleagues to estimate the AT by the almost universally used V-slope [12]. Clinically, this point is most commonly identified via a rapid incremental work-rate protocol on a cycle ergometer but it can also be achieved on a treadmill.

### Some opposition and repudiation

In 2011, Hopker *et al*. [22] expressed concern over not only the physiological mechanisms underpinning the noninvasive determination of the AT but also the clinical usage of the term. These authors suggested alternative explanations for the relationship between the conventionally estimated AT and surgical outcome. Whipp and Ward strongly repudiate much of this article, strongly supporting the conventional concepts of the AT [23]. The article by Hopker in turn quotes an article by Péronnet, which addresses the buffering mechanisms operating in exercise [24]. In their original article in 1964, Wasserman and McIlroy [11] had pointed out that the onset of anaerobic metabolism can be detected in three ways: (1) as an increase in the lactate concentration in arterial blood; (2) as a decrease in arterial blood bicarbonate concentration and pH; and (3) as an increase in the respiratory exchange ratio.

In 2012 Wasserman reiterated that the AT is the $\dot{V}O_{2}$ above which there is a sustained increase in arterial lactate concentration and the ratio of lactate concentration to pyruvate concentration during rapid incremental exercise [25]. Because of the accompanying hydrogen ion increase with lactate accumulation above the AT, and the buffer for the accumulating lactic acid being bicarbonate, CO_2_ output increases independently of O_2_ uptake above the AT. It was argued by Beaver *et al*., therefore, that the AT can be detected noninvasively at that point if there is no evidence of hyperventilation relative to CO_2_, which would be shown by an increase in the ventilatory equivalent for CO_2_, indicating hyperventilation [26].

**Figure 1:**
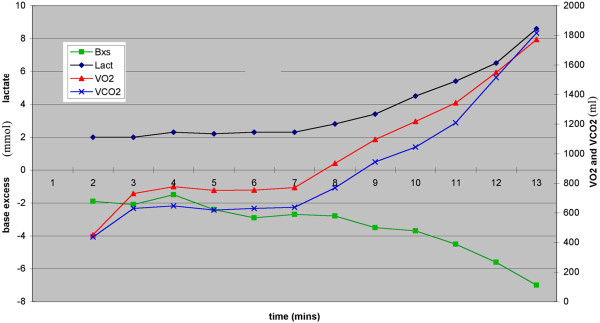
**Plot of base excess, lactate,**$V^{˙}{\;\mathrm{CO}}_{2}$**and**$V^{˙}{\;\mathrm{CO}}_{2}$**.** Simultaneous plot of lactate, base excess and gas exchange. This was obtained from a man exercising on a cycle ergometer. Samples at one minute intervals. Note that the base excess falls very much as the reciprocal of the lactate rise. Data from author’s laboratory.

Péronnet acknowledges that while there are many buffers in the muscle, bicarbonate is not the main buffer in muscle and that the fall in bicarbonate concentration does not mirror the rise in lactate concentration. The facts are that there is a delay in the fall of HCO_3_^−^ concentration until after the lactate concentration rises; after this the changes are very nearly equal and opposite [27]. Be this as it may, bicarbonate is the only buffer that releases carbon dioxide into the blood. The fall in arterial bicarbonate concentration that occurs *pari passu* with this buffering is accompanied by an increase in arterial lactate concentration that is very close to a mirror change, in fact almost a millimole to millimole change [21]. (Figure 1). Thus, at the time the arterial bicarbonate level is falling there will be an increase in CO_2_ output and a rise in arterial lactate level. If arterial blood can be sampled at very short time intervals, it will be seen that the rise in lactate level occurs a few seconds before the change in bicarbonate level, [16] reflecting a small contribution from non-CO_2_-yielding buffers [26]. Whatever other buffers are involved at this time they will not affect this outcome. This point at which the arterial lactate concentration increases indeed represents the onset of lactate-related anaerobic metabolism. The terms ‘lactic acidosis threshold’, ‘lactate threshold’ and ‘anaerobic threshold’ are all indicative of the same physiological point but reflect differing modalities of measurement. The moment in time that represents the onset of anaerobic glycolysis can vary slightly, that is reflecting the fact the rise in ${\dot{V}\mathrm{CO}}_{2}$ must occur after the decrease in arterial HCO_3_^−^ concentration; the CO_2_ has to reach the lungs in order to be removed! The temporal differences in metabolism will mean that the AT is identified at slightly different points.

This is not to argue against the importance of other buffering systems, but their function is important to the regulation of H^+^ concentration rather than additional CO_2_ production. If we believe that CO_2_ is a result of buffering of the lactate-associated proton by bicarbonate, then there will no increase in CO_2_ over that generated by aerobic metabolism, until ‘lactate acidosis’ becomes an issue. There can be a very slight increase in arterial lactate concentration at low intensity exercise but no fall in bicarbonate concentration or rise in $\dot{V}{\mathrm{CO}}_{2}$[15]. When the intensity of exercise increases, there will be a rapid formation of lactate at some point. The rapidity of this change will depend on many factors but it typically will produce a threshold-like increase in arterial lactate, which may take the form of a rapid to very rapid change, that is, a threshold, in the graph of lactate concentration against time. The rapidity of the change will depend on the rate of change of the work rate. It is important to remember that lactate is itself a fuel for muscle metabolism both locally during contraction and remotely in other muscle beds as well as the heart. The liver, during exercise, is not a major site of lactate clearance. Thus lactate is not reliant on one method of removal from the blood stream [19]. There is also a small amount of lactate as a pyruvate/lactate linked moiety, and all of these processes may slow the rate of change in arterial lactate [28]. Given that the source of this change results from an increase in anaerobic metabolism, then the term ‘anaerobic threshold’ is fully justified. This vindicates the use of the V-slope [14] method of determining the AT, which merely identifies this threshold point. It is important that hyperventilation, producing a rise in both ventilatory equivalents and a fall in end-tidal carbon dioxide tension, is not mistaken for the AT.

### Risk assessment of surgical patients

What then represents a reasoned approach to risk assessment of surgical patients using CPET? Aerobic capacity, measured as peak $\dot{V}O_{2}$ or AT, is reproducible and an objective reflection of physical fitness.

**Figure 2:**
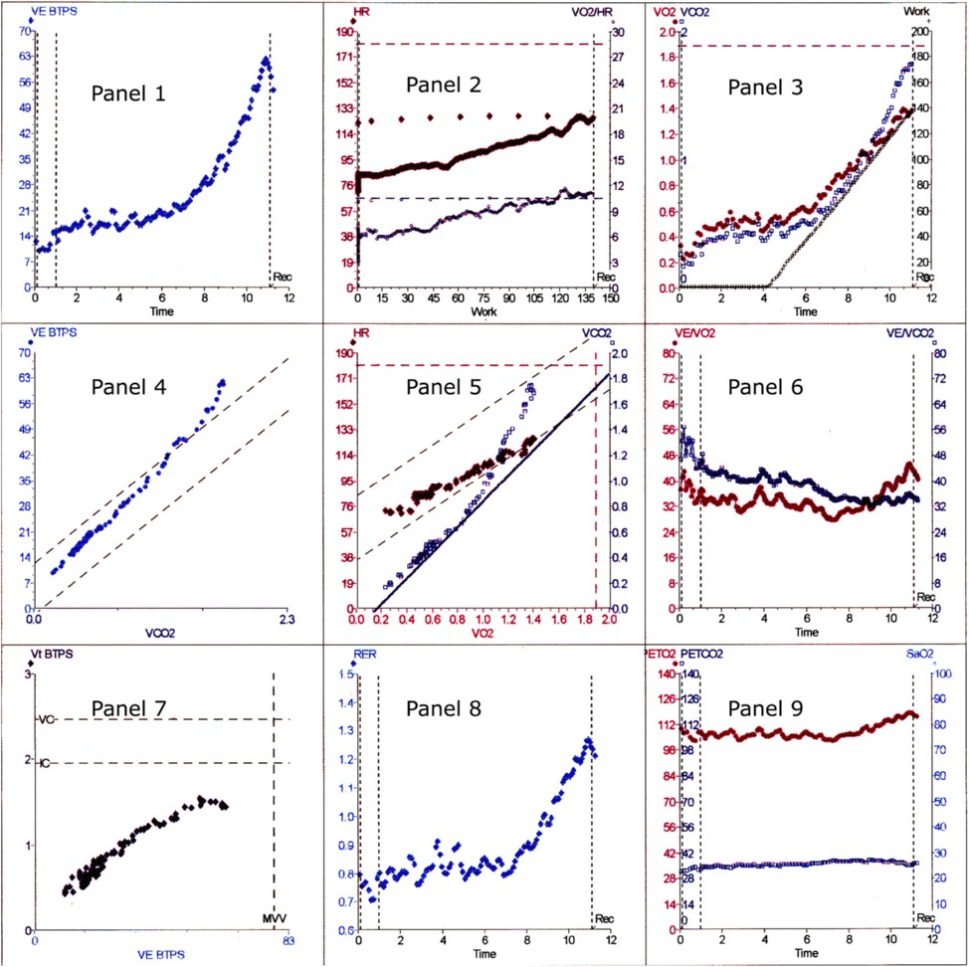
**Traditional nine-panel plot.** This format emanates from Wasserman and colleagues. This is one of several formats currently used but is by far the most common. There is a new format published recently from UCLA (Figure 3) and another one devised by Professor Whipp. The 9-panel format allows 15 variables to be plotted on 9 graphs. Note in Panel 7, the lines indicating vital capacity, inspiratory capacity and maximum ventilatory volume. This is useful in assessing respiratory function. *V*_e_, minute volume, *V*O_2_ = oxygen uptake, HR = heart rate, *V*CO_2_ = carbon dioxide output, *V*_t_, tidal volume, RER = respiratory exchange ratio, PETO_2_ = partial pressure end-tidal oxygen, PETCO_2_ = partial pressure end-tidal carbon dioxide. Data from author’s laboratory.

**Figure 3:**
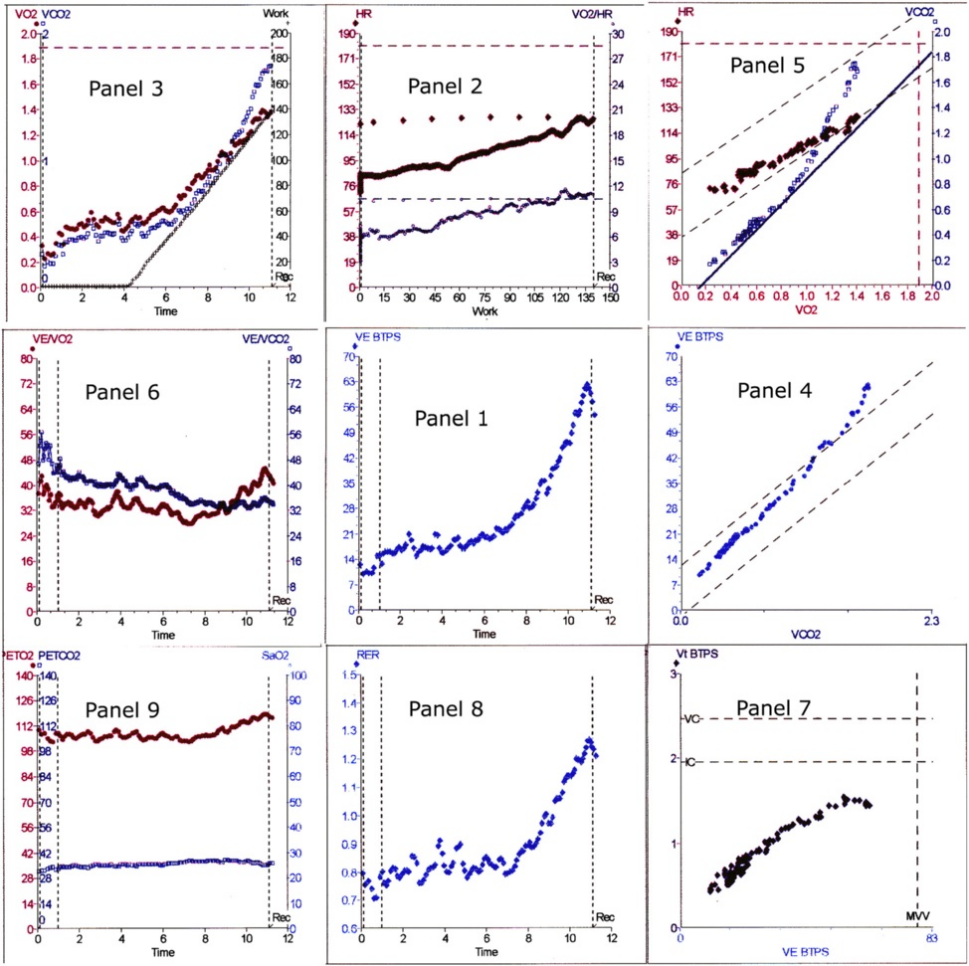
**New nine-panel plot.** This is the same test as Figure 2. The panel numbers refer to their original position in Figure 2. This new format was first announced in [16]. *V*_e_ = minute volume, *V*O_2_ = oxygen uptake, HR = heart rate, *V*CO_2_ = carbon dioxide output, *V*_t_ = tidal volume, RER = respiratory exchange ratio, PETO_2_ = partial pressure end-tidal oxygen, PETCO_2_ = partial pressure end-tidal carbon dioxide. Data from author’s laboratory.

The rapid incremental test evaluates cardiovascular (CVS), respiratory and peripheral muscle issues. The results of the test are traditionally reported in conjunction with the nine-panel plot advocated by Wasserman and colleagues (Figure 2); the most current version can be found in the fifth edition of *Principles of Exercise Testing and Interpretation* (Figure 3) [16]. This is a means of placing 15 variables onto 9 graphs and showing all the relevant results on one piece of paper. I will use this nine-panel format throughout this paper. The term ‘panel’ refers to this figure. This later plot uses ‘time’ on the *x*-axis in the three left panels (Panels 2,6,9).

Why is the V-slope so important? Almost every commercial metabolic cart, as a first option, estimates AT from the relationship between CO_2_ output and oxygen uptake, also termed the V-slope. (Figure 2, Panel 5) The computers may allow the operator to determine the AT or they may determine that point as part of the computer program. It is rare for the arterial lactate or bicarbonate levels to be actually measured. On its own, the V-slope is not a sufficient criterion for AT determination. Further confirmation of the AT should be sought from the ventilatory equivalent panel (Figure 2, Panel 6), and end-tidal gas tensions (Figure 2, Panel 9) to rule out nonspecific hyperventilation.

### Life after the AT

Many of the physiological responses to exercise undergo significant change above the AT. These include an increase in glycolysis resulting in increased lactate production, metabolic acidosis and increase in the $\dot{V}{\mathrm{CO}}_{2}$, with consequent increase in ventilation. These changes may translate clinically to an actual improvement in exercise competence, for example, an increase in O_2_ availability due to a shift in the oxyhaemoglobin dissociation curve [29] (the Bohr effect), allowing for greater tissue oxygen extraction. An important but detrimental effect is that exercise endurance is reduced. In simple terms, however, once over the AT the athlete may no longer be able to continue for an extended period as the build-up of lactic-acid-derived proton load and fall in pH occurs. There is a strong duration-intensity in this relationship in that the higher the intensity the shorter the duration of exertion. The fall in pH will inhibit muscle shortening velocity and inhibit the rate of glycolysis [30].

### Oxygen uptake: exercise vs. surgical stress

What has all this to do with the surgical stress response? While most of these physiological changes are described in relation to exercise many of the physiological changes are seen in patients following postoperative stress. Global oxygen uptake can increase by 50% following major surgery [31].

**Figure 4:**
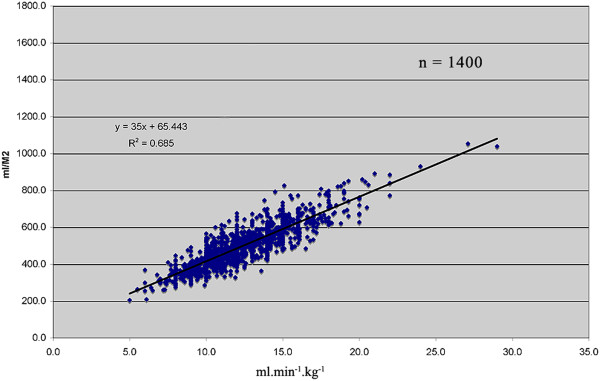
**Relationship of AT measured as ml.min**^**−1**^**.kg**^**−1 **^**to AT ml.min**^**−1**^**.m**^**2**^**.** The metabolic cart is usually programmed to index $V^{˙}O_{2}$ as ml.min^−1^.kg^-1^ whereas the pulmonary artery catheter is programmed to index as ml.min^-1^.m^2^. Note that ml.min^−1^.m^2^ indirectly uses height in indexing. Indexing as ml.min^−1^.kg^−1^ does not use height. This graph allows direct comparison. Data from author’s laboratory.

Some care needs to be exercised in interpretation of global oxygen uptake in exercise compared to global oxygen uptake in the postsurgical patient. Global oxygen uptake does not reflect the maximal oxygen uptake of a particular organ group; it is a weighted mean of all oxygen-consuming sites. Looked at from another perspective, the ceiling for oxygen consumption after surgery is lower than the ceiling during exercise. To further complicate the issue, postoperative $\dot{V}O_{2}$ calculated from cardiac output and arterial and mixed venous oxygen content from pulmonary artery catheter data is normally indexed as ml.min^−1^.m^2^ whereas the metabolic cart usually indexes $\dot{V}O_{2}$ as ml.min^-1^.kg^-1^. Note that this does not take into account the height of the patient. The correlation between these two approaches is shown in Figure 4.

Following surgery, the liver has a high oxygen demand and the muscle, whilst mechanically at rest, becomes a metabolic organ. Muscle is a site involved in the transamination of the branch chain amino acids, valine, leucine and isoleucine, and is thus able to convert glutamate from Krebs cycle to glutamine. Pyruvate is converted to alanine. Both glutamine and alanine, as well as lactate (C_3_H_5_O_3_^−^), are transported to the liver where C3 carbon skeletons are converted to glucose using free fatty acids as the energy source. This is termed the Cori cycle (after Carl Cori, Nobel Laureate in 1947). Lactate is produced in higher quantities than might be thought, as much of the postsurgical wound metabolism is lactate producing [19,32,33]. The liver has, at best, a parlous blood supply, as 75% of the blood supply is deoxygenated blood coming via the hepatic portal system. Under conditions of metabolic stress, the liver function may be compromised as the blood supply becomes inadequate. In the liver there is a segregation of function into zones of different metabolism. The periportal regions have differing functions from the perivenous regions. The periportal regions are gluconeogenic, whilst the perivenous are glycolytic [21]. This explains how the liver is capable of both net lactate release and gluconeogenesis from lactate. It is important that lactate can be metabolized at many other sites.

It follows that oxygen uptake response from an exercise test is not directly comparable to that in a postoperative patient. But in common with exercise, oxygen uptake postoperatively in major surgery is high.

### Preoperative cardiopulmonary status

In 1988 our group published the preoperative and postoperative cardiovascular system (CVS) status of 100 patients scheduled for major surgery [31] as determined by pulmonary artery catheter. The data in this paper agreed with the work of Del Guercio [34] and showed that in many instances the preoperative CVS status of many patients was poor yet frequently they had been passed as clinically fit for surgery. Evaluation of preoperative patients via a pulmonary artery catheter has many limitations, particularly in regard to pulmonary function. The main pulmonary function evaluated is the apparent intrapulmonary shunt at rest. The term CVS status is best applied to such evaluation. On the other hand, CPET evaluates cardiac, pulmonary and vascular function at rest and during exercise, thus justifying the term ‘cardiopulmonary’.

In our initial study cardiac index was 2.2 l.min^−1^.m^2^ or less in 11% of patients and a total of 20% had a cardiac index of 2.4 l.min^−1^.m^2^ or less. Intrapulmonary shunt equalled or exceeded 15% in 10% of patients. At that time, we stated that from the then recent work by Szlachcic [35], ‘it would appear that exercise $\dot{V}O_{2}$ may well detect patients who are unlikely to survive for 12 months because of their existing cardiovascular disease. To perform major surgery on this subset of patients is highly likely to lead to death early in the postoperative period.’ We felt that such methods of evaluation warranted more study in the surgical population. This then was the stimulus that made us investigate the cardiopulmonary status of preoperative patients by integrated CPET.

### The advantage of CPET evaluation of surgical patients

From the data that we had accumulated over many years it seemed that cardiac failure was the major cause of postoperative complications, not myocardial ischaemia. CPET could noninvasively measure and grade cardiac failure as well as detect myocardial ischaemia, both from the actual test as well as the electrocardiogram (ECG) [36]. For this reason, we felt that CPET was much more informative than an ECG stress test in assessment of operative risk analysis. More recently CPET has been demonstrated to predict all-cause postoperative mortality [37], to predict early mortality from cardiac failure in patients scheduled for heart transplantation [38] and to predict five-year survival after major surgery [39].

In 1999 our group published a prospective paper in which we analyzed 548 elderly patients who underwent CPET prior to major surgery [40]. All patients with an AT of <11 ml.min^−1^.kg^−1^ were admitted to the intensive care unit (ICU) preoperatively and monitored with a pulmonary artery catheter.

**Figure 5:**
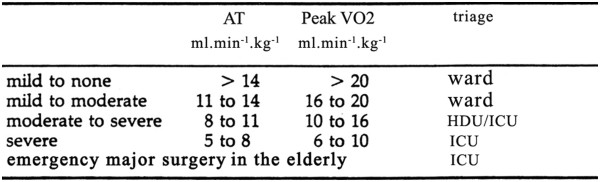
**Triage according to AT.** Patients with either no cardiac failure or mild to moderate cardiac failure are triaged to the ward. Only patients with moderate to severe or severe cardiac failure are triaged to ICU. Data modified from [42].

The papers of Weber, Janicki and colleagues [41,42] graded cardiac failure on the basis of both AT and peak $\dot{V}O_{2}$ (Figure 5). We chose to use the AT, as in our experience meaningful peak $\dot{V}O_{2}$ values are hard to obtain with consistency in the elderly. Our study results may differ from a hospital study performed today. Differences may include the level of care of postoperative patients on the wards, protocols of management within the ICU and experience in performing and interpretation of the exercise test.

CPET is not a therapeutic tool; it merely suggests the appropriate area of management based on operative risk. All patients with an AT lower than 11 ml.min^−1^.kg^−1^ were admitted by us to the ICU preoperatively and managed using pulmonary artery data. The same management applied to any patient defined as being in an area of ‘surgery-specific risk’ [43]. This policy resulted in 25% of all elderly patients having major surgery being admitted to ICU. If we were to perform this study today we also might find some different results, as postsurgical care should have improved over the intervening 12 to 13 years. Some critics of our paper have argued that the extra care lavished on the high-risk patients was a pivotal reason that they had such a low mortality. They may be right but if a little more care translates into a better mortality rate, the message is clear!

A further issue that became very important was that we were able to show that age was not a discriminator of mortality. This was highlighted by Wasserman in 1993 [44]. This needs to be borne in mind, as almost all ‘clinical’ discriminators of mortality rank ‘age’ as a key indicator of perioperative risk [45].

### Which variables are predictive?

To use AT values of exactly 11 ml.min^−1^.kg^−1^ as an indicator that the patient is fit to be managed on the ward, is to place too much emphasis on just one factor. Note that using the Weber and Janicki classification an AT greater than 11 ml.min^−1^.kg^−1^ but under 14 ml.min^−1^.kg^−1^ still indicates mild cardiac failure [31].

In our 1999 paper [40], we used both the AT and the ventilatory equivalent for oxygen $\left(\left(\dot{V}e/{\dot{V}O}_{2}\right)\right)$ as part of the predictive process. We also used the oxygen uptake to work rate ratio (the $\dot{V}O_{2}$ /WR slope) and the oxygen pulse but did not place predictive numbers on these variables.

**Figure 6:**
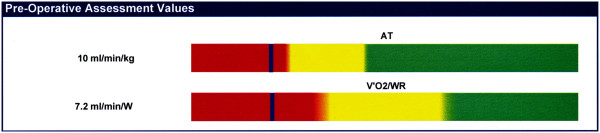
**Colour graphics interpretation of AT and **$V^{˙}{\;O}_{2}$**/WR slope.** This graphic shows the gradual change from high risk (red) to low risk (green) via an area of caution (yellow). It plots the AT and the $V^{˙}{\;\mathrm{VO}}_{2}$ /WR slope. This minimizes the assumption that an AT of 10.9 ml.min^−1^.kg^−1^ is very different from an AT of 11.1 ml.min^−1^.kg^−1^ (see text). Reproduced with permission from Cortex Biophysik GmbH.

I think I must take the blame for such a ‘black and white’ approach to the numbers and would urge the greater use of ‘shades of grey’. There is a thread of misunderstanding linking some of the large number of tests emailed to me for comment. Cardiac failure does not exhibit a stepwise deterioration over time. The deterioration is more a linear issue, thus an AT of 10.9 ml.min^−1^.kg^−1^ is not very different from an AT of 11.1 ml.min^−1^.kg^−1^; in fact the inter- and intra-rater variability might argue that they are the same. One manufacturer has developed a graphical display to show the AT and $\dot{V}O_{2}$ /WR lying on a coloured line that gradually changes from red to green via yellow (Figure 6). This may well be improved if the colours change from red to yellow through orange and from yellow to green through lime green.

Some studies do not find the AT as useful as the peak ${\dot{V}O}_{2}$[46-48]. This causes me no concern, as the peak $\dot{V}O_{2}$ is also a measure of aerobic capacity. The issue here is that the authors of these studies agree with our group, that aerobic capacity is the main factor correlating with survival rather than myocardial ischaemia. The largest study available confirming that heart failure is a greater surgical risk than myocardial ischaemia is that published by Hernandez *et al*. in 2004 [49].

I do have concern over the term ‘$\dot{V}O_{2\max}$’, as it is often taken as synonymous for peak $\dot{V}O_{2}$. The definition of $\dot{V}O_{2\max}$ is the highest $\dot{V}O_{2}$*attainable* by the subject despite increases in workload; anything less is the peak $\dot{V}O_{2}$[50,51].

### Where are we today?

#### The AT

Certainly a lot of attention needs to be paid to the AT (Figure 2, Panels 5, 6 and 9) but also to the ventilatory equivalents (Figure 2, Panel 6), the oxygen pulse (Figure 2, Panel 2), the $\dot{V}O_{2}$/WR slope (Figure 2, Panel 3) and ventilatory efficiency (Figure 2, Panel 4). The AT still measures surgical risk and even though the numbers may have changed over the years I would still stay with the numbers that we published in 1999 [40], at least until some more recent numbers are published. We could perhaps measure the AT and use a little more latitude in its interpretation. Perhaps an equation could be developed that used many of the CPET parameters to give a better risk analysis.

Generally, if the AT is less than 11 ml.min^−1^.kg^−1^, other parameters may well be abnormal. If, however, the AT were to be a little low, the surgery less stressful, for example a right hemicolectomy rather than a left, but the other parameters normal, the patient could go to the ward. This is not the place for a discussion of ‘Outreach’ but this is the sort of patient that could benefit from the attention of the ‘Outreach’ team [52]. The converse is easily managed. If the AT is a little greater than 11 ml.min^−1^.kg^−1^ but other parameters are abnormal, the patient should definitely go to the ICU. This is what I mean by ‘shades of grey’. It is safer to discharge someone from the ICU early than to admit someone to the ICU late!

If ventricular function is good then the increase in $\dot{V}O_{2}$ during exercise should be 10 ml.min^−1^.watt^−1^ or certainly better than 8 ml.min^−1^.watt^−1^, as most of our patients are sedentary (Figure 2, Panel 3). In some very fit patients the slope is over 11 ml.min^−1^.watt^−1^. This can cause some confusion. If the figure is 11 ml.min^−1^.watt^−1^ then might this imply a reduction in ‘efficiency’ as the subject is using more oxygen to achieve each watt than an elderly patient who has a slope of 8 ml.min^−1^.watt^−1^? The answer is elusive but it may result from utilization of different oxidative muscle fibres, utilization of additional muscle groups at high work rates or even an increase in the work of breathing at high minute volum. It does not imply anything sinister.

#### The oxygen pulse

The oxygen pulse should be near normal and the shape should be appropriate, that is, not an early low plateau. The ‘normal’ peak value of the oxygen pulse is the maximum predicted $\dot{V}O_{2}$ in millilitres, divided by the maximum predicted heart rate. Obviously, the use of different predictive nomograms will alter predictive values for other parameters including the oxygen pulse. If the peak $\dot{V}O_{2}$ is low but acceptable for the age of the patient then the oxygen pulse will also be reduced. The profile is as important as the actual value.

The oxygen pulse is also influenced by the haemoglobin via the C_(a – v)_O_2_:

$$\mathrm{Oxygenpulse}={\mathrm{VO}}_{2}/\mathrm{HR}=C_{\left(a–v\right)}O_{2}\times \mathrm{CO}/\mathrm{HR}$$

$$\;=C_{\left(a–v\right)}O_{2}\mathrm{xSV},$$

where C_(a − v)_O_2_ is arteriovenous oxygen difference, CO is cardiac output and SV is stroke volume.

The C_(a − v)_O_2_, the arteriovenous oxygen content difference, is influenced by the haemoglobin levels, such that a fall in haemoglobin concentration will result in a reduction in the C_(a − v)_O_2_ and thus a fall in oxygen pulse. To maintain the $\dot{V}O_{2}$, the stroke volume will need to be elevated.

It is possible to estimate the stroke volume at AT from the equation:

$$\mathrm{SV}=\left[O_{2}\mathrm{pulse}/C_{\left(a–v\right)}O_{2}\right]\times \mathrm{normalHb}/\mathrm{actualHb}$$

SV=9/0.1×150/120=9×10×150/120

SV≈120ml.

Assuming that C_(a − v)_O_2_ is 10ml/100 ml blood, Hb is 120 grams/litre, oxygen pulse is 9 and putative normal for haemoglobin is 150ml/litre

The C_(a − v)_O_2_ is (conveniently) approximately 10 ml per 100 ml of blood at the AT over a wide range of patients [53]. This measurement is difficult to make as it is very hard to be certain that the patient is at the AT when the arterial and mixed venous blood samples are taken.

**Figure 7:**
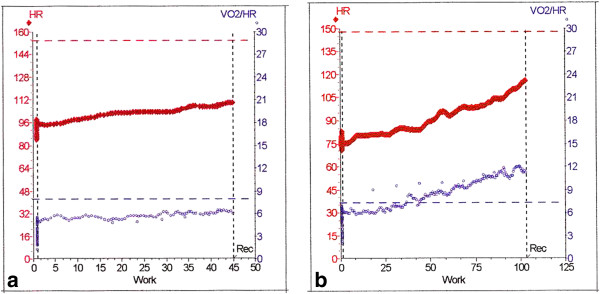
**Oxygen pulse under two different conditions. (a,b)** Panel 2 on the traditional Wasserman *et al*. 9-panel plot. In both cases, the patients are taking beta-adrenergic blocking drugs. (**a**) A low oxygen pulse in a woman. (**b**) An elevated oxygen pulse on a female patient. Note the low pulse rate and the compensating elevated oxygen pulse. The lower horizontal dotted line is the predicted value.

The oxygen pulse may cause some confusion when the patient is taking beta-adrenergic blocking drugs. It has been shown that the resting heart rate may not be indicative of the extent of beta-blockade [54]. The exercise heart rate is a far better guide. Bear in mind that the extent of the chronotropic response to beta-blockade is not necessarily the same as the extent of the inotropic response. If the heart rate is attenuated, the only way in which cardiac output can be increased is to increase the stroke volume, that is, an increase of the oxygen pulse over normal (Figure 7b). If this does not occur (Figure 7a), the cardiac output will be compromised and this will limit both the AT and the essential postsurgical response. It is the author’s view that this situation is an absolute indication of a need to review the beta-adrenergic blocking drugs if the patient is scheduled for major surgery. For a detailed discussion of beta-blocking agents and surgery see the POISE study [55].

#### The ventilatory equivalents

**Figure 8:**
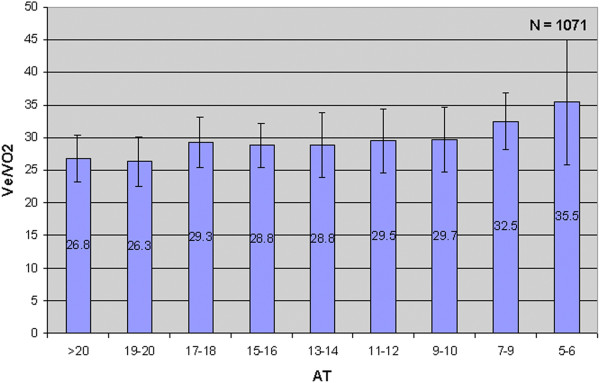
**AT ml.min**^**−1**^**.kg**^**−1 **^**vs.**$\;{\dot{V}}_{e}$**/**$\dot{\;V}O_{2}$**.** This graph shows how the ${\dot{V}}_{e}$ / $\dot{\;V}O_{2}$ rises as the value of the AT falls. A value for ${\dot{V}}_{e}$ / $\dot{\;V}O_{2}$ of greater than 35 is abnormal. Data from the author’s laboratory.

The ventilatory equivalents (Figure 2, Panel 6) are more of a problem. We have always viewed the ventilatory equivalent for CO_2_ (${\dot{V}}_{e}$ / $\dot{V}{\mathrm{CO}}_{2}$) as more of an indication of ventilatory function and have used the ventilatory equivalent for O_2_ (${\dot{V}}_{e}$ / $\dot{V}O_{2}$) as more related to the AT. Figure 8 shows the relationship of ${\dot{V}}_{e}$ / $\dot{V}O_{2}$. VO_2_ to AT of over 1,000 patients from the author’s laboratory. There is clearly a rise in ${\dot{V}}_{e}$ / $\dot{V}O_{2}$ as the AT falls. We feel that a value for the ${\dot{V}}_{e}$ / $\dot{V}O_{2}$ that is significantly outside this range, even if the AT is above 11 ml.min^−1^.kg^−1^, is an indicator that should guarantee high-dependency unit (HDU) admission. Providing that the ${\dot{V}}_{e}$ / $\dot{V}CO_{2}$ does not rise and the end-tidal oxygen tension does not fall, the nadir of the ${\dot{V}}_{e}$ / $\dot{V}O_{2}$ slope corresponds to the AT. If the AT cannot be identified by the V-slope method, then the nadir of the ${\dot{V}}_{e}$ / $\dot{V}O_{2}$ slope (Figure 2, Panel 6) is often quite usable as an alternative. We found that there was almost an 80% correlation between the AT derived by V-slope and ${\dot{V}}_{e}$ / $\dot{V}O_{2}$ nadir.

There is good evidence that the ventilatory efficiency reflects 'respiratory function and is shown by a ${\dot{V}}_{e}$ / $\dot{V}{\mathrm{CO}}_{2}$ slope of between 20 and 30 (over the linear region of the ${\dot{V}}_{e}$ / $\dot{V}{\mathrm{CO}}_{2}$ relationship, which normally extends upo the respiratory compensation point). This slope is usually very similar to the lowest ${\dot{V}}_{e}$ / $\dot{V}{\mathrm{CO}}_{2}$ ratio (Figure 2, Panel 6). The lowest ${\dot{V}}_{e}$ / $\dot{V}{\mathrm{CO}}_{2}$ value has been shown to be preferable as a noninvasive marker to the ${\dot{V}}_{e}$ / $\dot{V}{\mathrm{CO}}_{2}$ slope for the AT [56]. Abnormal ventilatory efficiency is associated with respiratory difficulties postoperatively and may become an indication for ICU management in the early postoperative period. It is preferable to admit such patients to ICU whilst still ventilated and extubate when everything else is finalized, for example, pain control.

**Figure 9:**
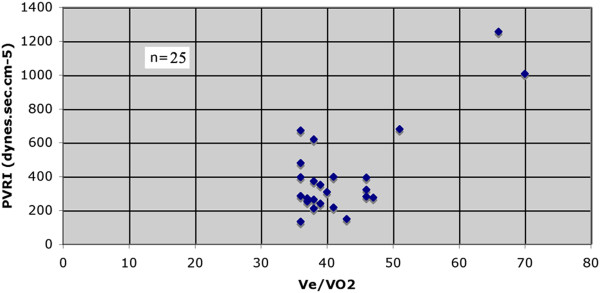
**Pulmonary vascular resistance vs.**${\dot{V}}_{e}$**/**$\dot{\;V}O_{2}$**.** All patients in this study had an AT of 10 ml.min^−1^.kg^−1^ or less. The graph shows the large variation in pulmonary vascular resistance with a ${\dot{V}}_{e}$ / $\dot{\;V}O_{2}$ of greater than 35. To be certain that the pulmonary vascular resistance will exceed 35 kPa.L^-1^.s, the V_e_ / $\dot{\;V}O_{2}$ needs to be greater than 45. Note the PVRI of those patients with a ${\dot{V}}_{e}$ / $\dot{\;V}O_{2}$ of 35. Data from the author’s laboratory.

As all our patients with a low AT were admitted to the ICU preoperatively and monitored by pulmonary artery catheters, we had an opportunity to investigate the relationship of the ${\dot{V}}_{e}$ / $\dot{V}O_{2}$ to the pulmonary artery pressures and pulmonary vascular resistance index (PVRI). The results were not quite as we expected. We studied 224 consecutive patients of whom 57 had an AT <10 ml.min^−1^.kg^−1^. Of these, 25 went to the ICU and on to surgery and thus we had access to pulmonary vascular data in this group of patients. We were able to compare these patients with those who did not have elevation of the ventilatory equivalents. We viewed all values for PVRI of over 35 kPa.L^-1^.s (350 dynes.s.cm^-5^)as of significance for postoperative care. We did not rely on the values for pulmonary artery pressures. Some patients following major surgery exhibit a fall in PVRI and others do not. We were unable to predict this response. We found that the ${\dot{V}}_{e}$ / $\dot{V}O_{2}$ needed to be over 45 to be certain that the patient has a PVRI > 35 kPa.L^-1^.s (Figure 9) It was worrying to find patients with a ${\dot{V}}_{e}$ / $\dot{\;V}O_{2}$ of 35 who had a PVRI of over 350 dynes.s.cm^−5^.

Any patient with a V_e_/ $\dot{\;V}O_{2}$ of 45 or greater should be investigated with a pulmonary artery catheter to establish the actual PVRI and cardiac output and then managed in the ICU.

## Conclusions

This paper may appear to have stopped in full flight. Maybe it has, because we certainly have not learnt how to garner all that we can from CPET. Calls have been made to develop a more ‘robust’ test than this for perioperative use [57]. I feel that what is needed is a greater ‘in-depth’ analysis of what we already have. CPET is not a ‘magic number’ and has basis in sound physiological principles.

Which patients should we test? If you think that only those with heart disease need testing, then there is no point in testing anybody! You already have a diagnosis in those who have heart disease and you will miss the rest, as they won’t be tested. Everybody over the age of 60 scheduled for major surgery needs testing. The AT is not a surrogate for physical fitness, as many people who regard themselves as ‘fit’ turn out on CPET to have significant but subclinical cardiac failure.

If you wish to be more precise, look at your hospital morbidity and mortality data and see the age at which ‘problems’ seem to be occurring.

Finally, it is mainly anaesthetists who will be carrying out these tests; they have a very different perspective of heart failure from cardiologists and you may well need their advice in certain areas. CPET is not intended to replace clinical examination or clinical judgement; it is designed to supplement them.

## Abbreviations

AT: Anaerobic threshold; C(a − v)O2: Arteriovenous oxygen difference; CO: Cardiac output; CPET: Cardiopulmonary exercise testing; CVS: Cardiovascular system; ECG: Electrocardiogram; HDU: High-dependency unit; ICU: Intensive care unit; PVRI: Pulmonary vascular resistance index; SV: Stroke volume; WR: Work rate.

## Competing interests

The author has received some remuneration from Cortex Biophysik GmbH for assistance with a computer program. This has no bearing on any material in this paper.

## Author’s information

Dr Older pioneered the use of CPET in surgical patients at the Western Hospital in Melbourne in the mid 1990s together with Dr A. Hall and Dr R. Smith. He was trained as an anaesthetist in the UK but later changed to Intensive Care Medicine. For twenty years he was Director of Intensive Care Medicine at the Western Hospital. Many anaesthetists have commented that being certain of the availability of an ICU bed affected how they managed the anaesthetic for their patient. His laboratory still operates today with Dr S. Frenkel and Dr R. Smith. Dr Older is currently a senior tutor in Medicine at Melbourne University and the CEO of the International CPET Society. He lectures widely internationally on CPET.
